# Interactions between Food and Drugs, and Nutritional Status in Renal Patients: A Narrative Review

**DOI:** 10.3390/nu14010212

**Published:** 2022-01-04

**Authors:** Claudia D’Alessandro, Alessia Benedetti, Antonello Di Paolo, Domenico Giannese, Adamasco Cupisti

**Affiliations:** Department of Clinical and Experimental Medicine, University of Pisa, Via Roma 56, 56126 Pisa, Italy; dalessandroclaudia@gmail.com (C.D.); alessiabenedetti98@gmail.com (A.B.); antonello.dipaolo@unipi.it (A.D.P.); domenico.giannese@phd.unipi.it (D.G.)

**Keywords:** diet, food, nutrients, medication, drug interaction, CKD, nutritional status

## Abstract

Drugs and food interact mutually: drugs may affect the nutritional status of the body, acting on senses, appetite, resting energy expenditure, and food intake; conversely, food or one of its components may affect bioavailability and half-life, circulating plasma concentrations of drugs resulting in an increased risk of toxicity and its adverse effects, or therapeutic failure. Therefore, the knowledge of these possible interactions is fundamental for the implementation of a nutritional treatment in the presence of a pharmacological therapy. This is the case of chronic kidney disease (CKD), for which the medication burden could be a problem, and nutritional therapy plays an important role in the patient’s treatment. The aim of this paper was to review the interactions that take place between drugs and foods that can potentially be used in renal patients, and the changes in nutritional status induced by drugs. A proper definition of the amount of food/nutrient intake, an adequate definition of the timing of meal consumption, and a proper adjustment of the drug dosing schedule may avoid these interactions, safeguarding the quality of life of the patients and guaranteeing the effectiveness of drug therapy. Hence, a close collaboration between the nephrologist, the renal dietitian, and the patient is crucial. Dietitians should consider that food may interact with drugs and that drugs may affect nutritional status, in order to provide the patient with proper dietary suggestions, and to allow the maximum effectiveness and safety of drug therapy, while preserving/correcting the nutritional status.

## 1. Introduction

Dietitians cannot prescribe or modify patient’s drug therapy, but the knowledge of patient’s history and medications (including herbal supplements) should be part of their professional practice. Drugs and food can interact mutually, since drugs can change the patient’s nutritional status, body weight and nutrient availability, while foods and herbal products may significantly influence the drug’s effects and efficacy in a direct or indirect manner: in the first case, a food or one of its components may modify the effect of the drug, in the second case, the food causes alterations in the absorption or metabolism of the drug.

Knowledge of these possible interactions is therefore fundamental for the implementation of nutritional treatment in the presence of pharmacological therapy. This is the case of chronic kidney disease (CKD), where nutritional therapy plays an important role in patient’s treatment, and changes in the nutritional status have a relevant prognostic significance. The actions and interactions of medications or supplements with food are extremely important in CKD patients, because the kidneys represent a common route of excretion; thus, some drugs need a dose adjustment on the basis of the residual kidney function. Moreover, old age, frailty and comorbidities are prevalent, and they may complicate the relationship between medications and food and vice versa. Therefore, mutual collaboration between nephrologists and dietitians is an essential prerequisite for a good clinical practice and nutritional treatment.

This review aims to investigate the possible interactions between drugs and nutritional status, and between food and drugs in CKD patients.

## 2. Interaction between Drugs and Nutritional Status

In CKD patients, the maintenance of a good nutritional status is a positive prognostic factor. Indeed, the prevalence of protein–energy wasting (PEW) increases with the worsening of the renal function and is associated with increased morbidity and mortality risk [1]. Moreover, obesity, especially the visceral type, is associated with an unfavorable prognosis.

The body composition status can be affected by the use of certain drugs, especially when they are administered chronically. For example, some of them may alter taste and smell, thus limiting food supply [2,3]. On the contrary, some drugs may stimulate appetite and food intake, resulting in an increased risk of overweight and obesity [4,5]. Moreover, some medications may have a direct effect on nutrients’ metabolism, mainly altering their absorption with the possible risk of deficiencies, especially for vitamins and minerals. Changes in the body composition may, in turn, alter the effects of a drug by changing the volume’s distribution. In addition, a recent systematic review showed that obesity could decrease the activity of CYP3A4/5, CYP1A2 and CYP2C9 cytochromes and increase the activity of CYP2E1. Weight loss seemed to be associated with an increased CYP3A4 activity, while CYP1A2 was not affected. A diet rich in carbohydrates or proteins reduced and increased the activity of CYP1A2, respectively. It emerged that body composition and diet may affect the enzymatic activity of cytochromes that play a major role in drug biotransformation [6].

### 2.1. Taste and Smell Abnormalities

Some drugs may induce dysgeusia which, in turn, can result in the loss of appetite and reduced food intake, potentially leading to PEW. Interestingly, dysgeusia can be caused by direct drug interaction with taste chemoreceptors, modification of the neurotransmitter function, alterations of action potential in nerve-cell membranes, changes in neural connections, or by the cytotoxic action of the drug [2,7]. Indirect mechanisms include the taste produced by the drug itself and an alteration of salivation. Ackerman and Kasbekar analyzed 1645 drugs and found that the 17% of them were associated with dysgeusia, and 61% with hypogeusia [2]. The classes of drugs that most frequently cause taste disorders are antineoplastic, immunomodulating, anti-bacterial agents and drugs acting on the central nervous system (CNS) [8,9,10,11].

It is worth noting that many taste disorders are associated with changes in smell (anosmia or hyposmia), which are less common and rarely reported by patients. The mechanism underlying this effect consists of a reduction in ion fluxes (Ca^2+^ and K^+^) or loss of ions (Zn^2+^) at the level of nervous and mucosal tissues (Table 1).

Alterations in taste and smell are often underestimated or considered not clinically important, but they have a negative impact on the quality of life and may affect nutritional status. In these cases, the patient tends to a spontaneous reduction in food intake; thus, reducing energy and nutrient intake, which may lead to undernutrition and a high risk of PEW.

### 2.2. Food Intake Changes

Drugs acting on the CNS can cause an increase or decrease in hunger, and therefore food intake which, physiologically, is finely regulated by neuropeptides, such as the alpha-melanocyte-stimulating hormone (α-MSH), or melanocortin, cocaine and amphetamine-regulated transcription (CART), leptin, ghrelin, neuropeptide Y and agouti-related protein (AGRP) and orexins. However, several neurotransmitters are involved in appetite regulation (i.e., dopaminergic, opioid, glutamatergic and GABAergic systems, as well as sympathetic and parasympathetic systems) [12,13]. Therefore, drugs acting on such systems may alter appetite, inhibiting or inducing it, and hence cause a decrease in or an excess in food intake, respectively [14]. Drugs that reduce appetite, so-called anorectic agents, include amphetamines, antidepressants and anti-diabetic medications as biguanides (metformin) and GLP-1 receptor agonists (liraglutide, semaglutide) [15]. In the recent past, many studies have focused on the anorectic effects of amphetamines and their derivatives, as well as active drugs affecting the endocannabinoid system, but the onset of serious adverse reactions (i.e., valvular heart diseases, psychiatric disorders) led to their withdrawal. On the contrary, antidepressant drugs (especially selective serotonin reuptake inhibitors, SSRI) can reduce food intake and have a safer pharmacological profile. These characteristics have contributed to the use of fluoxetine in patients affected by bulimia or compulsive overeating (Table 2).

It is worth noting that weight loss may be caused by the same mechanisms responsible for weight gain: the action of some drugs on the center of hunger can lead to the induction of satiety, reduction in food intake, and alterations to taste or smell that inhibit the desire for food and its palatability, as well as the nausea induced by certain medications (Table 2). Weight loss could be also the consequence of an increase in energy expenditure, or an alteration of hormones such as leptin that are involved in the regulation of carbohydrates and lipids metabolism.

The drugs that induce weight loss may act through three mechanisms: (a) reduction in nutrient absorption (i.e., fats in the case of orlistat); (b) reduction in food intake (i.e., bupropion, metformin, liraglutide); (c) increased energy expenditure (i.e., thyroid hormones).

As anticipated above, some widely used antidepressant medications such as SSRI may be labeled as anorectic drugs. Indeed, they act by blocking the reuptake of serotonin, dopamine and norepinephrine, promoting a good mood and decreasing the sense of hunger leading to an early onset of satiety, as bupropion does [16]. Bupropion received the approval from the Food and Drug Administration as an anti-obesity drug in combination with naltrexone [17]. However, other antidepressants such as tricyclic agents and other SSRIs (i.e., paroxetine and fluoxetine) may cause a minor weight gain (1–5 kg/year) [18]. Weight loss can also occur in people being treated with anticancer drugs that may have side effects such as loss of appetite, nausea, alteration of the oral and gastrointestinal mucosa, muscle weakness and asthenia [19,20].

Supplements represent another category of pharmaceuticals that should also be mentioned for their potential role in slimming practices. The use of supplements based on seaweed, rich in iodine, or other herbal principles is widespread and out of medical supervision. Ephedrine, pseudoephedrine, caffeine and theobromine may stimulate the thermogenesis [21]. Iodine is a stimulator of thyroid function, whereas guar gum, glucomannans and mucilage contain soluble fibers capable of stimulating satiety and slowing down digestion.

Corticosteroids and insulin act by increasing food intake; hence, they are included among medications that may induce weight gain, as well as antipsychotic drugs that reduce energy expenditure through the induction of sedative effects and alterations in the satiety control at the central level, even in pediatric patients [22].

Other mechanisms may be responsible for weight gain associated with drug administration, such as basal metabolism reduction through an action on thermogenesis, alteration of carbohydrates and lipids metabolism, changes in body fat distribution (mediated by an action on lipolysis and adipokines), and the induction of chronic fatigue and/or asthenia, a condition that leads to reduced physical activity and energy expenditure. Some examples are represented by alpha interferon, which can cause fatigue and decreased appetite, and penicillins [23], which are widely prescribed antibacterial drugs (i.e., ampicillin, amoxicillin, etc.) that are associated with several side effects, including weakness, asthenia and lack of appetite. Corticosteroids have a double effect: (a) they increase protein catabolism, leading to reduced muscle mass and loss of strength, while (b) stimulating appetite leading to an increase in body weight, namely due to fat mass increase and/or overhydration [24,25,26,27].

### 2.3. Changes in Resting Energy Expenditure

Resting energy expenditure (REE) represents approximately 55–70% of the energy requirement in healthy and physically active subjects. REE depends on age, gender, body composition, physical activity, health status and dietary habits, as well as on the percentage of lean mass and on visceral organs. Drugs active on the central control mechanisms (Pro-opiomelanocortin (POMC) neurons and orexigenic neuropeptides Y and AGRP), or peripheral ones (i.e., insulin, cortisol, thyroid hormones) can influence body weight by modulating carbohydrates, lipid and protein metabolism.

Steroidal anti-inflammatory drugs, estrogen-progestin preparations and antidiabetic drugs (both oral and injectable ones) affect glucose and/or fat metabolism [28]. Glucocorticoids, in particular, have multiple effects. They can induce insulin-resistance, as well as changes in fat-mass distribution, and water and salt retention through residual mineral corticoid activity.

Estrogens may also impair insulin sensitivity. Interestingly, estrogens promote liver synthesis, circulating HDL and triglycerides with increased uptake and peripheral metabolization of LDL, thus leading to an increased HDL/LDL ratio. Progestins show opposite effects, especially if they have a residual androgenic activity that can be marked weak (desogestrel and gestodene) or purely antagonistic (drospirenone and chlormalidone) [28].

Oral antidiabetic drugs have various mechanisms of action, so that they may induce a reduction in body weight (i.e., biguanids, GLP-1 agonists), an increase in body weight (i.e., glitazons, sulfonylureas) or have no effect on it (i.e., glinids, glitazons, DPP4 inhibitors). In addition, some of them are associated with a risk of hypoglycemia (sulfonylureas and glinides) as well as dysgeusia (biguanides) [29].

Finally, acarbose (an intestinal alpha-glucosidase inhibitor) and SGLT2 (type 2 sodium-glucose transporter) inhibitors may also be associated with weight loss.

Antipsychotic drugs of the first (i.e., haloperidol) and the second (i.e., olanzapine, clozapine and risperidone) generation affect glycemic control, both acting directly on the pancreatic β-cells and on the peripheral tissues inducing insulin resistance. In addition, the sedation effect leads to a reduction in energy expenditure and weight gain, particularly remarkable in adolescents. Antiretroviral protease inhibitors and nucleoside inhibitors of reverse transcriptase (NRTI) may favor weight gain by affecting pancreas β cells [30].

An alteration of glucose metabolism is also found during thiazides and beta-blockers use. The former may cause hyperglycemia by reducing insulin secretion secondary to hypokalemia, whereas the latter may induce hyperglycemia, and reduce peripheral insulin sensitivity and weight gain.

Table 2 shows the effect of some drugs or class of drugs on body weight.

### 2.4. Drug-Induced Nutrient Deficiency

Micronutrient (i.e., vitamins and minerals) deficiency is another condition of malnutrition, potentially induced by some medications interfering with absorption or excretion. The most common changes are those regarding potassium, sodium, magnesium, iron, calcium, zinc and copper. Indeed, some drugs can increase potassium excretion, and sodium retention, or reduce iodine uptake or release, reduce iron and zinc absorption and increase copper levels.

Hypokalemia is frequently associated with diuretics (loop and thiazide diuretics), β-adrenergic stimulants, or laxative agents [31], as well as some monoclonal antibodies used in oncology [32]. Hyperkalemia can also occur during therapy with renin-angiotensin-aldosterone system inhibitors (RAASi), namely aliskiren, ACE inhibitors, Angiotensin II receptor blockers (ARB), aldosterone receptor antagonists, β-blockers, nonsteroidal anti-inflammatory agents (NSAIDS), heparins, immunosuppressants (i.e., tacrolimus, cyclosporin), mineral corticoids and glucocorticoids, digoxin [33,34,35,36].

Hyponatremia is a common electrolytic disorder in people who take medications that act on sodium and water homeostasis, or increase the production/enhance the effect of the antidiuretic hormone (ADH), promoting the reabsorption of water at the level of the renal collecting tubule. Many medications may produce decreased sodium serum levels [37], including thiazide diuretics, SSRI, antipsychotics such as phenothiazine and haloperidol (which may cause an abnormal secretion of ADH), antiepileptics (i.e., carbamazepine, valproic acid), NSAIDS, proton pump inhibitors (PPI, namely omeprazole and esomeprazole), antineoplastics (i.e., vincristine and cyclophosphamide), and antidiabetics (i.e., chlorpropamide, tolbutamide).

A number of drugs may cause hypomagnesaemia [38]. Antibacterial drugs, such as tetracyclines, form an insoluble complex with metal cations; PPI and antacids lower the gastric pH and cause a down-regulation of the active intestinal transporter for magnesium TRPM6, whereas thiazide and loop diuretics prevent magnesium reabsorption at the renal level. Some antineoplastic agents (i.e., cisplatin) and birth control pills cause an increased renal excretion of magnesium. Finally, calcineurin inhibitors and iron-based phosphate intestinal binders are also associated with hypomagnesaemia [39].

Iron deficiency may be due to a reduced absorption, mainly caused by antibiotics such as tetracyclines and quinolones, and gastric antisecretory drugs, i.e., PPI and H2 receptor antagonists [40]. Indeed, gastric acid secretion facilitates the absorption of the free iron, allowing its conversion in the ferrous form that is more absorbable than the ferric one; hence, in reducing gastric acidity, the dietary absorption of this mineral is less efficient.

A condition of hypocalcemia may be the result of four different conditions [39,41]: hypoparathyroidism, hypovitaminosis D, calcium binding agents, or impaired bone resorption. Medications most often associated with hypocalcemia are loop diuretics (for increased calcium excretion), chelating agents (i.e., ethylenediaminetetracetate, citrate, phosphate), antineoplastic drugs (i.e., cisplatin, leucovorin, 5-fluorouracil, nab-paclitaxel, axitinib), biphosphates, calcitonin and denosumab (a monoclonal antibody used to treat osteoporosis).

Drugs that may facilitate copper and/or zinc excretion generally contain sulfhydryl groups, such as propylthiuracil and metimazole, captopril (an ACEi), and penicillamine (used in Wilson’s disease, rheumatoid arthritis, etc.) [42]. Interestingly, these drugs may cause also dysgeusia.

Common changes in vitamins availability may affect thiamine (B1), niacin (B3) and pyridoxine (B6), folate, together with vitamins B12, C and D.

Drugs that may cause vitamins B deficiency (in particular B12, B6 and B3) are mainly diuretics (they increase vitamins B removal, B1 in particular) and fibrates. Anti-acids as H2-antagonists and PPI may decrease vitamin B12 absorption by reducing gastric acidity [43]. Vitamin B12 deficiency may also occur with acetylsalicylic acid (ASA), antipsychotics (i.e., trifluoroperazine), colchicine, estrogens and metformin [44]. A reduction in vitamin B6 and vitamin PP levels may occur during treatment with antidepressants, particularly SSRI, and some antitubercular drugs (i.e., isoniazide). Folate deficiency may be caused by some antibiotics (penicillins, cephalosporins, tetracyclines), fibrates, birth control pills, ASA and antirheumatic drugs (namely Methotrexate), some chemotherapeutics, oral antidiabetics (in particular biguanides, and sulfonylureas), anticonvulsants (i.e., phenytoin, phenobarbital, primidone) and neuroleptics (phenothiazines).

Drugs able to cause vitamin C deficiency include diuretics, birth control pills and ASA.

Among the fat-soluble vitamins (A, D, E and K), the deficiency in vitamin D is the more prevalent, and may be caused by drugs such as statins, antacids, anticonvulsants (i.e., phenytoin), cholestyramine, glucocorticoids, and sevelamer, an intestinal phosphate binder [45].

Coenzyme Q10 (ubiquinone) is fundamental for the proper functioning of mitochondria. Some drugs can interfere with its function, as in the case of antidiabetic drugs (biguanides, metformin and in particular sulfonylureas glyburide and tolazamide), β-blockers, statins, corticosteroids, warfarin, and diuretics (i.e., acetazolamide).

## 3. Interaction between Food and Drugs

The possible interactions between food and medications are relevant in clinical practices [46], but often unknown or overlooked. They occur more frequently with orally administered drugs [47]. Indeed, food and beverages may alter the pharmacokinetic and pharmacodynamic profiles of a drug, leading to two different conditions:

(1) Increased concentrations in biological fluids that could enhance drug effect, up to the risk of side effects and toxicity;
(2) Reduced concentrations in biological liquids, and thus reduced effect of the drug, with the risk of total or partial ineffectiveness.

The first point to be considered is that interactions and their severity should be known and prevented, in order to avoid the risk of toxicity or therapeutic ineffectiveness. Indeed, although many interactions are hypothetical, some of them can be clinically evident, or can be classified as real adverse drug reactions (ADR). ADR are unintentional adverse reactions caused by a drug, and in the case of interactions with foods, they may have the characteristics of toxicity or therapeutic failure.

It is necessary to distinguish three factors that potentially increase the risk of serious interactions: the type of drug involved in the interaction, the severity of the disease for which the drug is administered, and the general conditions of the patient [20,48].

Drugs with a low therapeutic index can be involved in clinically evident interactions, due to the narrow range between efficacy and safety, since adverse events can appear at doses normally used for therapy. Hence, even small changes in blood or plasma concentrations of a drug with a narrow therapeutic range may result in toxicity or therapeutic failures. Immunosuppressive drugs (calcineurin or mTOR inhibitors), medications active on the cardiovascular system (antiarrhythmic drugs, cardioactive glycosides, oral anticoagulants) and on the respiratory system (i.e., theophylline) are examples of pharmacological agents with a low therapeutic index [49,50,51]. Drugs active on the central nervous system (such as antidepressants, anxiolytic-hypnotic, mood stabilizers, antiepileptics) may also be included in this group. Moreover, medications administered for chronic diseases expose patients to a greater risk of interactions because of the long-term administration.

The second factor is the severity of the disease for which the medications is required. For example, anti-coagulant therapy puts the patient at risk of hemorrhagic or thrombotic complications, in the cases of over-dosage or under-dosage, respectively [50]. Similarly, a patient may risk the rejection of the transplanted organ or the onset of toxicity in the case of an interaction with immunosuppressive drugs.

The third factor is identifiable in the general condition of the patient. For example, advanced age may be associated with cardiovascular or metabolic comorbidities, which may negatively affect drug–liver biotransformation and renal excretion [50]. The possible alteration of the absorption rate may be also caused by changes of the intestinal motility, a different body composition (i.e., reduction in fat tissue mass) and, above all, the reduced capacity of the organism in critical conditions to metabolize drugs and to eliminate them in stool or urine. The main food and drugs interactions are summarized in Table 3.

## 4. Pharmacokinetics Basis of Food–Drug Interactions

Pharmacokinetic interactions concern the processes of absorption, distribution, metabolism and elimination. In addition to single foods or nutrients, even the meal as a whole can significantly affect the pharmacokinetics of the drug, by changing its safety and therapeutic efficacy [52,53,54]. Moreover, the different pharmaceutical form in which the active ingredient is administered and the different chemical and physical characteristics, such as solubility or permeability along the gastrointestinal tract, may cause the drug to be differently affected by the food.

In general, among the four pharmacological processes, food can mainly interfere with absorption and metabolism. Indeed, the alterations of these two processes can change the effective bioavailability of the drug (i.e., the percentage of a dose of the active drug that is found in the body after its administration).

After an oral administration, the drug takes about 1–2 min to reach the stomach, where it dissolves, and part of the active ingredient passes in the bloodstream. The remaining part transits into the intestine, where the absorption is completed. The presence of a certain type of food may lead to a chemical–physical interaction, consisting in the formation of the molecular bond between the food and its component, and the active part of the drug. Consequently, this type of interference causes a decrease in drug absorption. Another mechanism is based on the modification of the physiology of the gastrointestinal tract [53], which occurs as a result of food ingestion, reduction in gastric acidity, increased gastric emptying times, changes in bile secretion, increased intestinal motility and alteration of the gut microflora composition. All of these alterations can finally change the absorption rate of a drug.

## 5. Changes of Drug Bioavailability

Drugs that are weak acid are absorbed at the stomach level, whereas weak bases are preferably absorbed at the small intestine level. Both chemical and physical properties may influence the absorption phase of drugs, such as the dissociation constant [55], a pH-sensitive parameter influenced by the formation of bonds and/or complexes with other molecular entities. Therefore, the introduction of a food which causes a change in the pH within the gastrointestinal tract, especially at the gastric level, can affect the ability of the drug to be absorbed. In addition, bonds and/or complexes may be formed between the drug and some molecules or ions contained in the food. Alteration of drug absorption by a food or a meal can also occur by binding of the active ingredient with the drug carrier protein; the competition between food and drugs for the binding with transport proteins can limit the absorption of the pharmacological agent.

Along the gastrointestinal tract, pH, perfusion, absorbent surface per unit volume and motility may influence the absorption rate of drugs in different ways [56]. For example, the gastric emptying time and the intestinal transit time are two factors that participate in the successful administration of the drug [57]. In particular, the ingestion of solid foods, especially if warm, viscous and rich in fat, causes a slowing of gastric emptying times and therefore a delay in the absorption of the drug at the intestinal level, even if the total amount of drug absorbed is unchanged. Furthermore, the ingestion of solid food stimulates the production of gastric and bile and pancreatic juices, which generally improves the dissolution of the drug and facilitate its absorption [58]. Meals with a high lipid content stimulate a greater production and release of bile in the duodenum, favoring a greater absorption of those drugs that need bile salts for an optimal absorption. Interestingly, some drugs conjugated with glucuronic acid undergo an enterohepatic circulation that ensures a longer presence within the bloodstream and the tissues [59].

## 6. Changes Due to Fluids, Protein, Lipid and Fibers Intake

Fluid intake can affect the absorption of a drug. Indeed, the volume and the temperature of beverages may alter the transit of the drug through the stomach, so modifying the time necessary for the appearance of the pharmacological effect [60]. With the exception of water, the intake of any beverage in conjunction with the drug could lead to a different absorption of the latter. Namely, there could be changes in the gastric pH, a delay of gastric emptying or chelation reactions, or the prevention of drug absorption. For example, this occurs with cola, cocoa, coffee (caffeine) and milk [61,62]. Moreover, the use of water for taking drugs prevents the adhesion of the drug to the esophagus and stomach wall, and allows a rapid transit to the absorption site. Attention should be paid to the fluid temperature: hot or too cold water should be avoided, because in both cases the gastric emptying time may increase.

Meal composition may affect the absorption of the drug in several ways. A high content in amino acids derived from high protein meal can form bonds with the drug or can compete with it for binding to the transport carriers [63]. Moreover, the increased secretion of pancreatic juices may cause an augmented quantity of water at the intestinal level, leading to drug dilution. On the other hand, a protein-rich meal increases blood supply to the intestine, facilitating and speeding up the absorption of the drug.

A meal rich in lipids delays gastric motility and increases bile production [64]. This kind of meal helps in the absorption of the so-called “lipophilic” drugs. On the contrary, the fiber content of a meal increases gastrointestinal motility, and reduces intestinal transit time. As a consequence, the bioavailability of drugs is reduced, together with their pharmacodynamic effects.

A special case of food–drug interference occurs between the intake of foods rich in tyramine (monoamine resulting from the amino acid tyrosine) and IMAO drugs, causing an excessive accumulation of monoamines that results in an acute increase in blood pressure and headaches. In this case, the diet should limit the intake of hard cheeses, beef, processed meat, yeast extract, dried fruit, soya, chocolate, etc. It is also important to pay attention to the intake of tyramine during treatment with linezolid, a drug used to treat severe infections. Indeed, linezolid acts as a MAO-inhibitor, so that its concomitant administration with foods rich in tyramine may cause a sudden increase in blood pressure.

Another important factor is the time between the administration of the drug in respect to the meal or food intake [65]. The drugs most sensitive to these interactions are mainly those unstable in gastric fluids or that are more likely to form bonds with food molecules. For this reason, the drugs are divided into two main categories: drugs that must be taken with a meal or in conjunction with a meal (administered in the half hour before or after the meal) and drugs that must be taken without food, namely about 2 h before or 3–4 after the meal.

## 7. Changes of Drug Distribution

Several factors influence the volume and speed of drug distribution, depending on the tissue in which the drug is distributed and the chemical–physical characteristics of the drug, so that the percentage of fat and lean mass of the subject affects the rate of distribution of a drug, its half-life and the time needed to reach the steady state, in both adult and pediatric patients [66].

The pharmacokinetics of the lipophilic drug is mainly dependent on the adipose body mass. Fat tissue is poor in water (especially intracellular), and scarcely vascularized. Lower water content causes a lower distribution of hydrophilic drugs in the adipose tissue, and a greater distribution of lipophilic ones [67]. On the contrary, hydrophilic drugs administered in overweight or obese subjects, who have a higher percentage of adipose body mass, are distributed in a lower volume of water. Therefore, the calculation of the dose based on actual body weight may expose the subject to the risk of overdosing, because the quantity of fluids in which the drug is distributed is not proportional to body weight. As a result, the drug will have a higher plasma concentration and longer half-life, and presumably greater effects than expected, even if that rule may not be true for all drugs [68,69].

An additional factor influencing drug distribution is represented by the binding with plasma proteins, because only the free form of the drug can spread within the extra-vascular space or within the cells where it exerts its effects. Of note, albumin binds acidic drugs, whereas acid alpha-glycoprotein and lipoprotein basic drugs, combined with several factors (i.e., liver and renal diseases, inflammation, cancers), may affect the concentration of plasma proteins available for drug binding [70,71].

Changes in body composition occur not only in the case of overweight/obesity, or lean-body-mass reduction, but also in extreme age ranges. Indeed, both in newborns and the elderly, body composition is quite different than in adults. In newborns there are high percentages of body water (75–80% approximately) and low percentages of fat mass, whereas in the elderly there are physiological reductions in body water and increase in adipose tissue [72,73]. With advancing age, there is also a lower binding capacity of plasma proteins (namely, hypoalbuminemia), a lower plasma volume, a reduced enzymatic activity and a decreased kidney function. This leads to an alteration in the distribution volume of drugs: in older patients, water-soluble drugs have a lower volume of distribution, while fat-soluble drugs have a higher volume of distribution in respect to body weight.

## 8. Changes in Drug Metabolism

It is worth noting that drugs, nutrients and food may affect the activity of liver enzymes, resulting in an increased or decreased metabolism of drugs, and consequently in a diminished or augmented pharmacological effect, respectively. The CYP450 isoforms (namely, CYP3A4,5,6, CYP1A2, CYP2B6, CYP2C8, CYP2C9, CYP2C19, CYP2D6 and CYP2E1) are the most important enzymes involved in drug biotransformation.

In particular, the intake of certain foods (i.e., soy) or beverages (i.e., grapefruit and blueberry juice) may inhibit the activity of cytochrome P450 enzymes, thereby altering the concentration of the drug at the level of the target site of action [74]. In the case of grapefruit juice, the inhibition lasts for several hours and hepatic CYP3A4 activity returns to normal within 48 h from the intake of the juice [75]. It is clear that the risk of a food–drug interaction, which could be clinically relevant, may depend on the safety of the drug (i.e., therapeutic index), the duration of the concomitant intake of both the drug and the food, and, finally, on the clinical conditions of the patient and the severity of the disease. Furthermore, a diet that is high in protein and lipid content but poor in carbohydrates is capable of inhibiting the CYP450 activity, and consequently of increasing the plasma concentration of the drug. This inhibition is particularly severe for long, unsaturated chains of fatty acids [76].

## 9. Changes in Drug Elimination

The kidney is primarily responsible for the elimination of most drugs, through glomerular filtration and tubular secretion. It is worth noting that the free fraction of the drug that is filtered by glomerulus can, however, be rapidly reabsorbed at the tubular level, if it is in the non-ionized form. The weak acid or base character of the drug explains the equilibrium between the dissociated and undissociated form that is dependent on the pH of the ultrafiltrate, and consequently the final excretion or the reabsorption of the dissociated or undissociated forms of the drugs, respectively. Therefore, all those foods or beverages that can acidify or alkalize the urine, can then alter the resorption and facilitate the excretion of certain medications. An example are the so-called alkalizing or acidifying diets [77]. An alkaline diet is characterized by the presence of vegetables and fresh fruit and a reduced intake of acidifying foods; it is rich in sulphur, phosphorus and chlorine, contained in foods such as cheese, meat, sausages, eggs, simple sugars, refined flours, coffee and tea. On the contrary, in the acidifying diets, the intake of proteins of animal origin is high, and low in fruit, vegetables and pulses (the so called western diets). The acidity or alkalinity of a food can be defined by the PRAL index (Potential Renal Acid Load): foods with negative PRAL are potentially alkalizing, whereas those with positive PRAL are acidifying [77]. There are also foods that are “neutral”, or slightly acidifying, such as whole grains, legumes, milk and dried fruit.

## 10. Pharmacodynamics and Pharmacokinetics of Food–Drug Interactions

An analysis of the scientific literature showed that scarce attention is addressed to the investigation of food–drug interactions. Moreover, food contains so many compounds capable of interfering with drugs that it is difficult to investigate all of them [78]. Another explanation is that the concentrations of food constituents and nutrients capable of altering drug pharmacodynamics depend on multiple variables, such as the type of fruit/vegetable, the geographical origin, the harvest season, the degree of maturation of the fruit/vegetable, and storage conditions [79]. For this reason, most studies have been focused mainly on food supplements and drinks, or extracts such as fruit juices, teas, herbal teas, alcoholic beverages, coffee and milk.

## 11. Vegetables Rich in Vitamin K

Among the best-known drug–food interactions, the association of warfarin with foods rich in vitamin K is certainly the best known. The warfarin effect is due to the incomplete synthesis of coagulation factors, through the γ-carboxylation of glutamic acid residues, for which vitamin K plays an essential role. Foods rich in vitamin K may interfere with the therapeutic effect of the drug. These foods are mainly represented by crucifers (broccoli, cabbage, etc.), lettuce, spinach, parsley, etc. High vitamin K content may be found also in asparagus, peas, lentils, soy, egg yolk, liver etc. Despite this risk, patients on warfarin treatment may eat those vegetables, paying attention to eat a moderate quantity over a long time, eating the same amount daily, and adjusting the warfarin dose consequently. A recent metanalysis reported that the restriction of vitamin K intake does not seem to be a useful strategy to improve the efficacy of warfarin [80]. Several studies have found a negative relationship between vitamin K intake and variations of the international normalized ratio (INR), while others have found a positive, but dose dependent, relationship: with a minimum intake of vitamin K, it is still possible to maintain adequate anticoagulation effect. If the intake exceeds 150 μg/day of vitamin K, the effect of the drug is altered [80]. Therefore, a useful approach to overcome this problem is to maintain a stable dietary habit, avoiding wide changes in vitamin K intake [80,81].

## 12. Goitrogenic Foods

Another well-known food–drug interaction is between levothyroxine and so-called goitrogenic foods, which include crucifers (cabbage, cauliflower, broccoli, etc.), soya, lettuce and spinach, milk and some additives such as nitrites. These foods can interfere with the metabolism of iodine that is essential for the proper activity of the thyroid gland through the synthesis of T3 and T4 thyroid hormones [82]. Indeed, the high concentration of isothiocyanates in these foods can inhibit the incorporation of iodine and therefore the formation of thyroxine, decreasing thyroid function [83]. However, in the majority of cases, this does not imply the complete exclusion of these foods from the diet. It is possible to consume them, paying attention to the amount, frequency and time of consumption. In any case, patients may have these foods occasionally, in moderate servings and not earlier than 30–60 min from the intake of levothyroxine.

However, some of these interactions have not been confirmed, as in the case of soya that may increase the risk of hypothyroidism. A recent systematic review has shown that eating soya does not affect thyroid hormones, and may result in a modest increase in thyroid stimulating hormone (TSH) levels [84]. Therefore, in the context of a varied diet, it is possible to consume soya in subjects with thyroid problems, provided that the diet is not deficient in iodine. Special care should be taken in the case of Hashimoto thyroiditis treated with levothyroxine, because soy can interfere with this drug. However, a soybean intake at least 4 h away from the drug can be considered harmless [84].

## 13. Fruit or Vegetable Juices

Grapefruit, orange, apple, pomegranate, blueberry and tomato juices have been investigated for their potential interactions with medications. Among all fruit juices, grapefruit juice is the best known [85]. In fact, it is a potent inhibitor of the activity of some isoforms of the cytochrome P450 active in the intestine, CYP3A4 isoform in particular, which is responsible for the detoxification of about 50% of drugs.

This inhibitory activity is due to some substances contained in the grapefruit and its juice, namely the naringine (phenolic compound with anti-inflammatory and antioxidant properties) and the bergamottine (furanocumarin). The list of medications that can be affected by grapefruit juice is long, and includes commonly prescribed medications such as [86]:

- Statins (i.e., atorvastatin);
- Antihypertensive drugs such as calcium-blocker agents (amlodipine, felodipine, manidipine, nicardipine, nifedipine, nimodipine, nisoldipine, nitrendipine, pranidipine, etc.), angiotensin II receptor antagonists (losartan), and β-blockers (thalnol and acebutolol);
- Immunosuppressant agents (cyclosporin and tacrolimus);
- Antiarrhythmic (amiodarone, quinidine, disopyramide and propafenone);
- Antineoplastic (vinblastine);
- Antibiotics (erythromycin).

Flavonoids contained in grapefruit juice, such as naringinine and hesperidine, are responsible for the inhibition of transmembrane transporters, which play a role in the passage of the drug from the intestinal lumen within the bloodstream. These compounds are also present in other fruit juices, such as citrus fruit juices. Indeed, orange, apple, kiwi and papaya juices, which contain the same flavonoids (naringine, hesperidine, and floridzin, floretin) are able to inhibit the transport polypeptides of organic anions (OATP) at the usual doses. Namely, 1–2 fruits of standard size or 200 cc of commercial or homemade juice are enough to inhibit this process [79,87,88,89]. The intake of these fruit juices determines the reduction in gastrointestinal absorption of certain antibiotics, antihypertensive, beta-blocker and antiallergic drugs. In particular, the coadministration of drugs such as acebutolol, celiprolol or fexofenadine with grapefruit juice, or atenolol, ciprofloxacin, and fexofenadine with orange juice, decreases the oral bioavailability of antihypertensive and anti-histaminergic drugs [90].

Among the drugs carried by the OATP system, the most important is levothyroxine; it is mandatory to pay attention to the concomitant intake of citrus fruits with this drug, although other studies have shown that vitamin C seems to increase its absorption. The discrepancy of these results is an indicator of a variability of interference, where the reduction in absorption is dependent on the dose. Nevertheless, this interaction may become clinically evident, so that the usual recommendation is to avoid taking levothyroxine within 3–4 h from the consumption of citrus fruits or fruits in general.

Studies have shown that blueberry juice [91] and pomegranate juice [92] can inhibit the CYP3A and CYP2C9 isoforms. Blueberry juice, in particular, has proven to be capable of interacting with warfarin. Indeed, this juice contains several flavonoids that can play the role of inducers or inhibitors of CYP450 and, in particular, the CYP2C9 isoform [93]. This enzyme is involved in the metabolism of warfarin, so the concomitant intake of blueberry juice can lead to an abnormal increase in INR, and therefore bleeding risk.

Pomegranate juice has been shown to interfere with medications such as antidepressants, anti-hypertensives (especially the class of ACE inhibitors) and anti-inflammatory drugs. Indeed, three components of pomegranate juice (namely, ursolic, oleanolic and gallic acids) were able to inhibit the transmembrane transporters; thus, preventing pharmacological molecules from entering into the cells and interacting with their molecular targets [94].

Some studies suggested that aloe juice could reduce the effectiveness of some chemotherapeutic drugs, although it may increase the effect of oral antidiabetics due to a further decrease in blood glucose levels when aloe juice is taken with those drugs. The aloe juice should not be taken with medications such as thiazide diuretics, glucocorticoids and cardioactive glycosides, to avoid the risk of increased renal potassium excretion leading to hypokalemia [95].

Pineapple juice or its extracts can interact with NSAIDs, warfarin, antiplatelet agents and heparin, causing an increased risk of bleeding. Vegetable juices such as cabbage, onion and green pepper juices have proven to be able to competitively inhibit CYP3A4 activity [93]. However, those inhibitory effects were not tested in vivo, and the number of flavonoids contained in those vegetables is dependent on the growing conditions, so it is not possible to conclude with certainty that cabbage and onion can inhibit CYP3A4 activity at a clinical level [94]. Tomato juice contains one or more competitive direct inhibitors of CYP3A4 activity [96]. This effect has also been observed in other solanaceous plants, such as potatoes, eggplants, and peppers; therefore, it is believed that these vegetables share the same inhibitory compounds [97].

Overall, these studies bring further important information. First, fresh or homemade juice is less likely to inhibit drug uptake than commercial juice. Second, it has been illustrated that the reduction in drug uptake is directly proportional to the amount of juice consumed and to the time between juice and drug intake [87]. In general, it has been observed that a period of time of four hours between the consumption of juice and the intake of the drug is recommended to avoid any chance of interaction [87].

These studies have several limitations, the most important one being that they have been carried out in vitro. Few studies have investigated drug–foods interactions in vivo, and rarely in humans [91]. The short exposure to food and nutrients, usually lasting two weeks, could explain the lack of clinical trials [98,99].

## 14. Alcohol

Alcohol may interact with drugs because it is metabolized in the liver by cytochromes P450 [100]. The interaction may lead to an increased concentration of alcohol and/or drugs in the blood stream, resulting in an increase in the deleterious damage to the central nervous system, especially when the drugs belong to the classes of antidepressants, anxiolytics, antiepileptics and hypnotics. Indeed, alcohol consumption may increase the sedative effect of some drugs, induce mood changes, reduce alertness and judgment, and decrease muscle tone, up to occurrence of coma [101].

Other interactions may occur with the concomitant intake of analgesics (NSAIDs and paracetamol), antibiotics (cephalosporins), antihistamines, antihypertensives (ACE inhibitors, diuretics, beta-blockers), bronchodilators, statins, anticoagulants and sulfonylureas.

Moreover, alcoholic beverages such as wines and beers contain several flavonoids and polyphenols that could be responsible for food–drug interactions. Among these substances, trans-resveratrol and gallic acid in red wine, and phenolic acids and prenylflavonoids in beer have proven to be potential inhibitors of cytochrome CYP activity [75,102].

The interaction between alcohol and disulfiram is clinically useful to reduce alcohol consumption. Disulfiram inhibits aldehyde dehydrogenase that converts acetaldehyde derived from alcohol into acetic acid. By reducing that biotransformation, the increasing concentrations of acetaldehyde (up to 5–10 times higher than normal) causes symptoms including accelerated heartbeat, a reddened and swollen face, low blood pressure, abdominal cramps, nausea, vomiting, headaches and hypoglycemia. Interestingly, that effect can also occur with other drugs, such as metronidazole, cephalosporins, sulfonylureas, chloramphenicol, griseofulvin, and quinacrine, which manifest a “disulfiram-like” reaction [103,104].

## 15. Tea

The content of catechins (especially epigallocatechin) in tea could be responsible for the inhibition of OATP transmembrane transporters, even if that effect was not clinically evident in human trials [78]. The consumption of green tea is not recommended in subjects taking antibiotics, anticoagulants, oral contraceptives, anxiolytics, chemotherapeutic or antihypertensive agents, because catechins can inhibit the OATP1A2 transporter in the intestinal mucosa. Therefore, this affects the absorption of several drugs, as well as nadolol (an antihypertensive beta-blocker), rosuvastatin (statin), chemotherapy agents (erythromycin, levofloxacin, imatinib, methotrexate), antivirals (saquinavir) and other agents (i.e., rocuronium, fexofenadine, thyroxine) [51,105,106]. However, clinical studies of possible interactions between tea and drugs have been carried out on a limited number of subjects [78].

## 16. Coffee

The scientific literature reports possible interactions between the intake of coffee, or beverages containing caffeine (cola drinks, cocoa), and some drugs. Indeed, the caffeine found in several beverages and food is able to interact with different medications in different ways. For example, concomitant intake of caffeine-containing beverages/foods may reduce the effectiveness of hypnotics, drugs for the treatment of urinary incontinence (such as anticholinergics, topical estrogens) and lithium. In other cases, caffeine enhances the effects of the drug, leading to the onset of side effects. For example, caffeine increases: (a) the effect of oral warfarin, increasing the risk of bleeding; (b) the stimulation on the sympathetic nervous system by MAO-inhibitors, leading to episodes of cardiac arrhythmias or hypertension; (c) the gastric damaging effects of NSAIDs [107].

Another important interaction occurs with the intake of clozapine, an atypical antipsychotic drug, whose serum concentrations may rise by 20–26% due to the inhibition of the CYP1A2 isoform by the caffeine contained in a 40 mL cup of coffee [107]. On the contrary, the interaction between caffeine and felodipine, which was demonstrated during in vitro studies, was not observed in clinical trials. An important interaction occurs when bronchodilator drugs containing theophylline are administered together with caffeine, because both molecules can accelerate breathing and heart rate, so their concomitant intake is not recommended.

Finally, the concomitant intake of some medications (i.e., oral contraceptives, quinolone antibiotics) may increase serum levels of caffeine which may accumulate, leading to signs and symptoms such as nausea, vomiting, nervousness, anxiety and tachycardia [108]. In any case, it is important to add that the pharmacokinetics of caffeine are highly variable among individuals, due to polymorphism at the level of the CYP1A2 isoform of cytochrome P450, which metabolizes 95% of the caffeine ingested. So, the entity of interaction with the same drug may change among individuals [109].

## 17. Milk

Milk may interfere with some antibiotics, reducing the bioavailability of tetracyclines and fluoroquinolones when administered per os. Indeed, these drugs may form complexes with calcium present in milk and dairy products that can seriously compromise their absorption [78].

## 18. Macronutrients: Proteins and Lipids

It is well known that the efficacy of medications could be modified when taken per os within the meal, because food components and bioactive compounds may interfere with each other.

Research has mainly focused on understanding the chemical mechanisms underlying these interferences. Indeed, high-energy and high-fat meals are more likely to modify gastrointestinal motility and the intestinal absorption of drugs [110]. In particular, high-fat meals may reduce serum concentrations of cycloserine (an antitubercular drug) and esomeprazol (a PPI), or increase the absorption of fat-soluble drugs such as saquinavir and atazanavir (antiretroviral protease inhibitors) [111]. Protein-rich meals can potentially increase serum albumin levels, and/or cytochrome P450 activity and alter the effectiveness of warfarin (as demonstrated by the reduction in the INR ratio). Similar consequences were observed in the case of levodopa, because amino acids such as isoleucine, leucine, valine, phenylalanine, tryptophan and tyrosine compete for the transmembrane transporter that allows drug absorption within the intestinal mucosa; as a result, the bioavailability of the drug is reduced, as is its pharmacological effect. On the contrary, the bioavailability of propranolol is increased when assumed with foods, resulting in an increase in its effects [76].

## 19. Fibers

A fiber-rich meal may alter the absorption of some drugs, depending on the fiber-induced increased gut motility, although studies are scarce and results may be contradictory, due to individual variability [112]. Simvastatin, ezetimibe, pravastatin and fluvastatin may suffer a reduction in efficacy when assumed in conjunction with a high-fiber intake. Accordingly, the concomitant intake of pectin or oat bran and lovastatin may reduce drug absorption.

## 20. Food Supplements

The potential risk of drug interactions exists also for food supplements, because they may contain active molecules (naturally contained or added during industrial processes) which are associated with adverse events. For these reasons, food supplements are generally safe for the general population, but are not completely risk-free for medical patients [113]. Some components of food supplements may be pharmacologically active, or act as enzyme inhibitors or inducers, being capable of altering the pharmacokinetics and the effects of drugs [114]. Many studies investigated the interactions between supplements and drugs to overcome the lack of awareness about the potential drawbacks of these products and to motivate caregivers to ask their patients about the use of supplements [115]. The topic is becoming increasingly prevalent, given the growing and widespread consumption of these products in recent years; it is estimated that about 32 million Italians, aged 35–64 years and mostly women, consume food supplements. About 18% of them bought a supplement on the advice of family, friends, the internet, TV, or magazines.

In general, reports on the adverse events attributable to herbal or phytochemicals products are quite rare; the effects are generally mild and include headaches, nausea, and fatigue. More serious events, such as liver damage or bleeding, have also been described [114,116].

The difficulty in obtaining information on food supplements and drug interaction is represented by the study methodology. Most information comes from preclinical models, whereas the gold standard should be represented by clinical trials. The aim is to identify the interactions, the target enzymes (i.e., CYP isoforms) and transmembrane transporters (i.e., ABC and SLC families) by co-administration of an enzyme-specific probe. Moreover, some natural compounds contained in supplements have been shown to activate nuclear receptors, such as PXR (Pregnane X Receptor) and CAR (Constitutive Androstane Receptor) [117]. These receptors regulate the expression of a set of genes involved in the bioactivation, detoxification and transport of endogenous and exogenous substances, including drugs [114].

## 21. Herbal Products

The use of herbal products is common worldwide; unfortunately, there are no efficacy data for many of these compounds and no quality safety regulations, so that “natural” does not always mean “safe”. Indeed, many of these products are identified with more than one name, and sometimes reflect the plant from which they derive [118,119]. Furthermore, the dose is not regulated and sometimes the composition is not clear; information about the purity of the products is scarce, and very often there are contaminations from heavy metals [120]. Furthermore, herbal compounds such as *Hypericum perforatum*, *Ginkgo biloba*, and *Salvia chinensis* and preparations such as Yin zhi huang (a Chinese herbal product) are able to activate nuclear receptors, such as PXR and CAR [117].

Among the various compounds that were found to interfere with some drugs, the most known is the *Hypericum perforatum*, a natural antidepressant remedy also called Saint John’s worth. The active ingredient is hyperphorin, which is able to inhibit neurotransmitters such as serotonin, norepinephrine, dopamine, glutamate, γ-aminobutyric acid. Moreover, *Hypericum* can modulate the activity of CYP3A4, CYP2C9 and CYP1A2 isoforms and P glycoprotein, because hyperphorin binds the nuclear receptor that regulates the expression of intestinal CYP3A4 and glycoprotein P.

Therefore, *Hypericum perforatum* can interact with a number of medications [121], reducing their degradation and hence increasing their circulating levels and effects:

- Immunosuppressants (cyclosporin, tracrolimus);
- Anticancer drugs;
- Oral contraceptives (ethinyl estradiol, norethindrone, ketodesogestrel);
- Cardiovascular drugs (anticoagulants, statins, beta-blockers);
- Antimicrobials (including anti-HIV, voriconazole);
- Antidepressants and anxiolytics (benzodiazepines and buspirone);
- Anticonvulsant agents;
- Oral hypoglycaemic agents (tolbutamide, glycazide);
- Anaesthetics;
- Respiratory (fexofenadine) and gastrointestinal (omeprazole) agents;
- Anti-migraine drugs (eletriptan);
- Muscle relaxant medications;
- Medications used in drug abusers (i.e., methadone).

*Ginkgo biloba* is another import herbal compound that has been proven to interfere with the effectiveness of some medications [122]. It is used to improve brain performance and reduce fatigue. It is also used in cases of Alzheimer’s disease and *laudication intermittens*, as it can improve cognitive functions in cerebrovascular diseases and peripheral blood circulation [123,124]. The substances contained in the *Ginkgo biloba* that have pharmacological properties are flavonoids and tripertenic lactones (gingkoloids and bilobalids). As a result, *Gingko biloba* is able to reduce platelet aggregation, and acts on the CPY2C9 and CYP3A4 isoenzymes, inhibiting the microsomial metabolism of warfarin. Therefore, herbal preparations containing *Ginkgo biloba* should be avoided in patients treated with antiplatelet or anticoagulant drugs [125,126].

The inhibitory action of Ginkgo extract on cytochrome P450 isoforms seems to be responsible for other pharmacokinetic interactions involving the calcium-channel blocker nifedipine and the PPI omeprazole. Other studies have shown that flavonoids contained in Ginkgo can exert an agonist action with the GABA system and a serotoninergic effect, interacting with benzodiazepine and SSRI, respectively [127,128,129].

Senna preparations (*Cassia angustifolia* and *acutifolia*), based on its natural cathartic properties, has the potential to interact with drugs [130]; hence, they should be avoided in association with drugs that may induce hypokalemia, as well as thiazide or loop diuretics, corticosteroids, or with cardiac glycosides, antiarrhythmics, and β-blockers [131,132]

## 22. Liquorice

Liquorice is made from rhizomes and roots of the plant named *Glycyrrhina glabra*. Its excessive consumption can lead to increased arterial blood pressure and hypokalemia [133,134]. This effect is attributable to the metabolite glycyrrhizin acid that reduces the hepatic and renal metabolism of corticosteroids by inhibiting the 11-beta-hydroxysteroid-dehydrogenase enzyme. Consequently, the aldosterone-like activity of cortisol at the renal level increases, inducing pseudo primary hyperaldosteronism, resulting in hypertension, hypokalemia, metabolic alkalosis and hydro-saline retention. The consumption of this preparation should be avoided during antihypertensive therapy, or with drugs that could cause hypokalemia (i.e., thiazides or loop diuretics, and corticosteroids) [130,135].

## 23. Ginseng

Ginseng includes numerous species of the *Araliaceae* family. This root is used to obtain different products, such as a coffee substitute drink and supplements. It has antioxidant, antipyretic, cholesterol lowering, anticancerogenic, anti-inflammatory properties and it is believed to improve memory function. For this reason, it is used in the case of hypotension, diabetes, gastritis, insomnia, fatigue, and mental stress [136,137]. The use of ginseng is not harmless, even if the intestinal metabolites of its constituents (ginsenosides) on which the beneficial properties depend, may strongly inhibit the cytochrome-dependent metabolism and transmembrane transporters [138,139]. Therefore, consumption of ginseng is not recommended together with certain medications such as anticoagulants (warfarin), phenelzine (IMAO), some chemotherapeutics (imatinib), oral hypoglycemic and insulin, digoxin, anticonvulsant (lamotrigine) and antiestrogenic therapies [140,141,142].

## 24. Spices

Spices may also interfere with a drug’s metabolism. Recently, a group of Japanese researchers investigated the action of 55 spices on the cytochrome system [143]. Interestingly, cinnamon, black and white pepper, ginger, turmeric and nutmeg may inhibit the activity of CYP isoforms 3A4 and 2C9, which catalyze the biotransformation of many drugs so that their effects are prolonged. For example, turmeric increases the effect of chemotherapy drugs, whereas cloves increase the action of antibiotics. On the contrary, ginger can increase the gastric discomfort of NSAIDs. However, the interactions can be more often detected when spices are taken in high doses, and the drugs have a lower therapeutic index/narrow therapeutic range, as in the case of oral anticoagulants.

## 25. Black Pepper

Piperine contained in black pepper inhibits both P-glycoprotein and cytochrome CYP3A4 [144,145], so it can modify the concentration of those drugs that are the substrate of both systems. In particular, P-glycoprotein recognizes as substrates certain chemotherapeutics (etoposide, doxorubicin, vinblastin), digoxin, immunosuppressants, glucocorticoids (dexamethasone), anti-HIV agents, colchicine, tacrolimus and quinidine. Substrates of CYP3A4 include tricyclic antidepressants (amitriptyline, clomipramine, imipramine), benzodiazepine (alprazolam, midazolam, triazolam), antibiotics (erythromycin, clarithromycin, dapsone), antihistamines (terfenadine, astemizole), calcium-channel blockers (nifedipine, felodipine, diliazem, verapamil), cyclosporin, lovastatin, dexamethasone, carbamazepine, cisapride, ethinyl estradiol, glyburide [143].

## 26. Turmeric

The curcumin contained in the rhizome of the *Turmeric longa* has been shown to inhibit the activity of both the transmembrane transporters belonging to the ABC family and CYP isoforms [146]. Therefore, curcumin may alter the pharmacokinetics of drugs that are substrates of those proteins (i.e., cardiovascular agents and drugs acting on central nervous systems) [146,147,148,149].

## 27. Ginger

Zingiber officinalis, or ginger, has been shown to be a natural anticoagulant molecule. It also reduces blood pressure and modulates glucose serum levels. For this reason, people who use oral anticoagulants or anti-platelet drugs, and diabetics on insulin treatment, should be avoid the assumption of ginger [130,150].

## 28. Chili

Chili peppers can interfere with the intake of certain medications, due to its active ingredient capsaicin. This alkaloid has many properties (i.e., analgesic, soothing, antibacterial, antioxidant, anticancer, antidiabetic properties) and it may be useful in the case of obesity, as it seems to increase thermogenesis [151,152,153]. Unfortunately, some studies have shown the possibility that chili pepper may cause drug interactions, especially when consumed with drugs acting on the cardiovascular system, because capsaicin has a vasodilating action. For this reason, its association with drugs such as aspirin, clopidogrel, diclofenac, naproxen, ibuprofen, oral anticoagulants (warfarin) and heparins should be avoided [151,154].

## 29. Cinnamon

Cinnamon has also been attributed multiple properties, among which includes the reduction in plasma levels of glucose, antioxidant and neuroprotective activity. The most important drug interaction is with antidiabetic drugs, such as glimepiride, insulin, pioglitazone, rosiglitazone, chlorpropamide, glipizide and tolbutamide. As cinnamon powder can potentially lower glucose plasma levels, it can act in synergy with antidiabetic drugs, leading to hypoglycemia risk. Cinnamon also contains coumarin and cinnamaldehyde, which have an inhibitory activity against some CYP isoforms [155], and may interfere with anticoagulants, enhancing their effect and the risk of bleeding [156,157].

## 30. Conclusive Remarks

**Figure 1:**
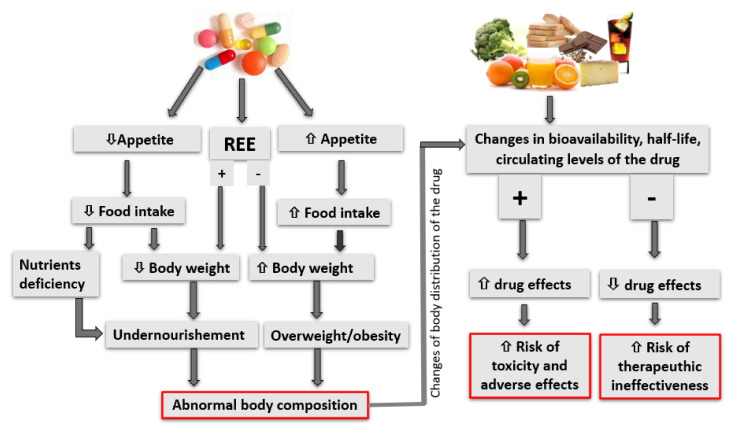
A graphical synthesis of the possible interactions between drugs and the nutritional status, and between food and drugs in CKD patients. ⇧ = increase; ⇩ = reduce.

The present paper aimed to collect, in a single document, the interactions that may take place between drugs potentially used in renal patients, as well as the nutritional status of different food and nutrients (Figure 1). The molecules contained in different quantities of food can interfere with the pharmacokinetics and pharmacodynamics of drugs. Due to this relevance, a given patient’s pharmacological history deserves particular attention, and must be part of the dietician interview. It is a common finding that patients informed about a possible food–drug interaction immediately avoid certain foods or nutrients. However, this incorrect or even wrong behavior may lead to nutrient deficiency and unnecessary limitations. A proper definition of the amount of food/nutrient intake, an adequate definition of the timing of meals consumption, and a proper adjustment of the medication dose may avoid interactions without affecting the quality of life of the patients, and guarantee the effectiveness of drug therapy. This result may be obtained through a close collaboration between the nephrologist, the renal dietitian and the patient.

Therefore, dieticians, nephrologists and physicians should be aware of:

- The existence of possible drug–food interactions, with some drugs influencing nutritional status;
- The pharmacological therapy of the patient, comprehensive of supplements and herbal medications;
- The need to educate the patient of a correct diet, in order to maximize the effectiveness and the safety of drug therapy while preserving/correcting nutritional status.

Those recommendations would increase the efficacy of pharmacological treatments while sparing patients from unwanted drug effects.

## Figures and Tables

**Table 1:** Medications stimulating food intake.

Amitriptyline, imipramine (tricyclic drugs) and mirtazapine (an atypical antidepressant)
Duloxetine and venlafaxine (serotonin and norepinephrine reuptake inhibitor, SNRI)
Citalopram and fluvoxamine (selective serotonin reuptake inhibitor, SSRI)
Isoniazide, iproniazide, nialamide, phenelzine (monoamine oxydase inhibitors, MAOI).
Clozapine, olanzapine, quetiapine and risperidone (Second-generation neuroleptics);
Valproic acid, lamotrigine, gabapentin, pregabalin and vigabatrin (Antiepileptic drugs)
Oxandrolone and prednisone (Androgenic steroids)

**Table 2:** Drug and nutritional status interaction. Mechanisms underlying nutritional status changes are discussed in the text.

⇧ Body Weight		⇩ Body Weight	
Antidepressant	mirtazapine, fluvoxamine, phenelzine, citalopram amitriptyline, doxepin, imipramine, trimipramine	Anticancer	
Neuroleptics	clozapine, olanzapine, risperidone, quetiapine, haloperidol	Drugs to treat obesity and diabetes	orlistat, metformin, liraglutide, GLP1 agonists
Corticosteroids	cortisone, prednisone, oxalandrone	Amphetamines and derivatives	phenifuramine, phentermine
Benzodiazepines	alprazolam, diazepam, clonazepam	Antipsychotics	phenothiazines
Estrogen progestin	levonorgestrel	Psychostimulants	methylphenidate, glucagon
Antiepileptics	valproic acid, gabapentin	Immunomodulators	lenalinimide, aldesleukin, interferon alfa
Antidiabetics	glibenclamide, glicazide, repaglinide, pioglitazone	Antihypertensives	captopril, diltiazide, nifedipine, reserpine
Interferon alfa		Nasal decongestants	oxymetazoline, nafazoline
Penicillins	ampicillin amoxicillin	Orexin antagonists	suvorexant, lemborexant
Antiretrovirals	indinavir, ritonavir		
Antituberculous	isoniazide		

⇧ = increase; ⇩ = reduce.

**Table 3:** Food–drug interactions. Foods to avoid or not to take in conjunctions with medications, due to the risk of severe interference with drugs.

Food	Drug or Drug Classes	Interactions
Grapefruit, juice and fruit	Amiodarone, amlodipine, antihistamines, atorvastatin, carbamazepine, carvedilol, cyclosporine, diazepan, disopyramide, erythromycin, ethinylestradiol, losartan, repaglinide, sertraline, simvastatin, stomach acid-blocking drugs, tacrolimus, thyroid replacement drugs, triazolam, verapamil	⇧ Effect of drugs metabolized by cytochrome P450
Hypericum or St. John’s wort	Anticoagulants, antidepressants, anti-proteases, beta-blockers, cyclosporine, oral contraceptives, digoxin, immunosuppressants, statins, theophylline	⇧ Drugs metabolism lowering therapeutic effectiveness
Alcoholic beverages	Anxiolytics, antidepressants, antiepileptics, antihistamines, opioids	Concurrent consumption ⇧ sedative effect or can cause paradoxical effects
Caffeine	Anxiolytics, antidepressants, medications for insomnia and urinary incontinence	⇩ The action
	Anticoagulants, clozapine, MAOIs, NSAIDs, theophylline	⇧ The effect
Green leafy vegetables	Anticoagulants (warfarin, acenocoumarol)	Source of vitamin K Alteration of drug effects with sudden changes in their intake
Liquorice	Antiarrhythmics, antihypertensives, diuretics, digoxin,	Salt and water retention, hypokalemia
Ginkgo Biloba	Warfarin	⇧ risk of bleeding
Milk and dairy	Antibiotics (ciprofloxacin, tetracycline)	⇩ Absorption
Orange, kiwi, papaya, and apple juice	Antihistamines, celiprolol, ciprofloxacin, fexofenadine	⇩ Absorption
Hard cheeses	IMAO, linezolid	Source of tyramine, risk sudden increase in blood pressure
High protein intake	Levodopa	⇩ Absorption
Leagy vegetables, soy, cabbage	Levotiroxina	Inhibition of iodine incorporation
Ginseng	Antidepressant	Induction of manic episodes
Garlic, blueberry juice	Warfarin	⇧ Risk of bleeding
Chocolate	Sertraline, paroxetine, fluoxetine, fluvoxamine, citalopram	⇧ Effect

⇧ = increase; ⇩ = reduce.
